# XGBCDA: a multiple heterogeneous networks-based method for predicting circRNA-disease associations

**DOI:** 10.1186/s12920-021-01054-2

**Published:** 2022-11-03

**Authors:** Siyuan Shen, Junyi Liu, Cheng Zhou, Yurong Qian, Lei Deng

**Affiliations:** 1 School of Software, Xinjiang University, Wulumuqi, 830091 China; 2 School of Computer Science and Engineering, Central South University, Changsha, 410075 China

**Keywords:** Association prediction, circRNA, XGBoost

## Abstract

**Background:**

Biological experiments have demonstrated that circRNA plays an essential role in various biological processes and human diseases. However, it is time-consuming and costly to merely conduct biological experiments to detect the association between circRNA and diseases. Accordingly, developing an efficient computational model to predict circRNA-disease associations is urgent.

**Methods:**

In this research, we propose a multiple heterogeneous networks-based method, named XGBCDA, to predict circRNA-disease associations. The method first extracts original features, namely statistical features and graph theory features, from integrated circRNA similarity network, disease similarity network and circRNA-disease association network, and then sends these original features to the XGBoost classifier for training latent features. The method utilizes the tree learned by the XGBoost model, the index of leaf that instance finally falls into, and the 1 of K coding to represent the latent features. Finally, the method combines the latent features from the XGBoost with the original features to train the final model for predicting the association between the circRNA and diseases.

**Results:**

The tenfold cross-validation results of the XGBCDA method illustrate that the area under the ROC curve reaches 0.9860. In addition, the method presents a striking performance in the case studies of colorectal cancer, gastric cancer and cervical cancer.

**Conclusion:**

With fabulous performance in predicting potential circRNA-disease associations, the XGBCDA method has the promising ability to assist biomedical researchers in terms of circRNA-disease association prediction.

## Introduction

CircRNA is a covalently closed loop structure [1], and its downstream 5’ splice site is connected to the upstream 3’ splice site [2]. In recent decades, the researches regarding circRNA have entered into a stage of rapid development. Emerging evidence indicates that plenty of circRNAs are related to critical biological processes. Among these processes, one of the significant aspects is the associations between circRNA and diseases, with the gradually increasing numbers of circRNA-disease associations verified by biological experiments. Jelenia et al. discovered that circRNA plays a paramount role in the evolvement of cancer. Specifically, their study manifested that cancer-related chromosomal translocations cause fusion circRNA(f-circRNA), and F-circRNAs show tumor-promoting effects in vivo models [3]. Wang et al. conducted a study showing that heart-related circRNA(HRCR) is an antihypertrophic molecule that can inhibit cardiac hypertrophy and heart failure by targeting miR-233 and ARC [4]. Liu et al. detected a new circRNA involved in the process of cartilage damage, and further proposed that circRNA-CER may be used as a potential target for osteoarthritis OA [5]. Moreover, circRNA also has a close relationship with bladder cancer, colorectal adenocarcinoma, esophageal squamous cell carcinoma, lung adenocarcinoma and other cancers [6–9]. Although circRNA has become a marker for the diagnosis of specific diseases, traditional experiments cost substantial time and resources. Thus, a fast and economical method to detect the connection between circRNA and human diseases is of great significance.

To start the analysis of the association between circRNA and diseases, it is necessary to establish a circRNA database first. Currently, multiple databases storing circRNA information have been constructed. The circBase database collects information such as the sequence, gene and genome location of circRNA and its latest update was in July 2017 [10]. The Circ2Traits database is the first disease-circRNA association database [11]. The CircNet database accumulates expression profiles, genome annotations and sequences of circRNA subtypes, and provides circRNA-miRNA gene regulatory networks [12]. The CircR2Disease gathers experimentally verified circRNA-disease associations and contains 725 associations between 661 circRNAs and 100 diseases in its latest version [13]. The CircInteractome database includes a search function for possible interactions between circRNA and RBP and miRNA [14]. The exoRBase database visualizes the collection of circRNA, lncRNA and mRNA derived from the analysis of human blood exosomal RNA-seq data [15]. The CSCD database developed by Xia et al. is designed to study the function of cancer-specific circRNA [16].

There are many methods proposed to predict circRNA-disease associations. For example, Deng et al. predicted circRNA-disease associations based on the KATZ method and the integration between circRNA, protein and disease [17]. Lu et al. proposed a method for predicting circRNA-disease associations based on sequence and ontology representations of convolutional neural networks and recurrent neural networks [18]. Li et al. used a deep learning method called DeepWalk to extract features, and then used a network consistent projection method for circRNA-disease association prediction [19]. Wang et al. used stacked autoencoders to extract features, and carousel forest (RF) classifiers for circRNA-disease association prediction [20]. Zheng et al. proposed the iCDA-CGR model to predicate circRNA-disease associations based on chaotic game representation [21]. Wang et al. proposed a calculation method based on multi-source information combined with deep convolutional neural network (CNN) to predict circRNA-disease association [22].

**Fig. 1:**
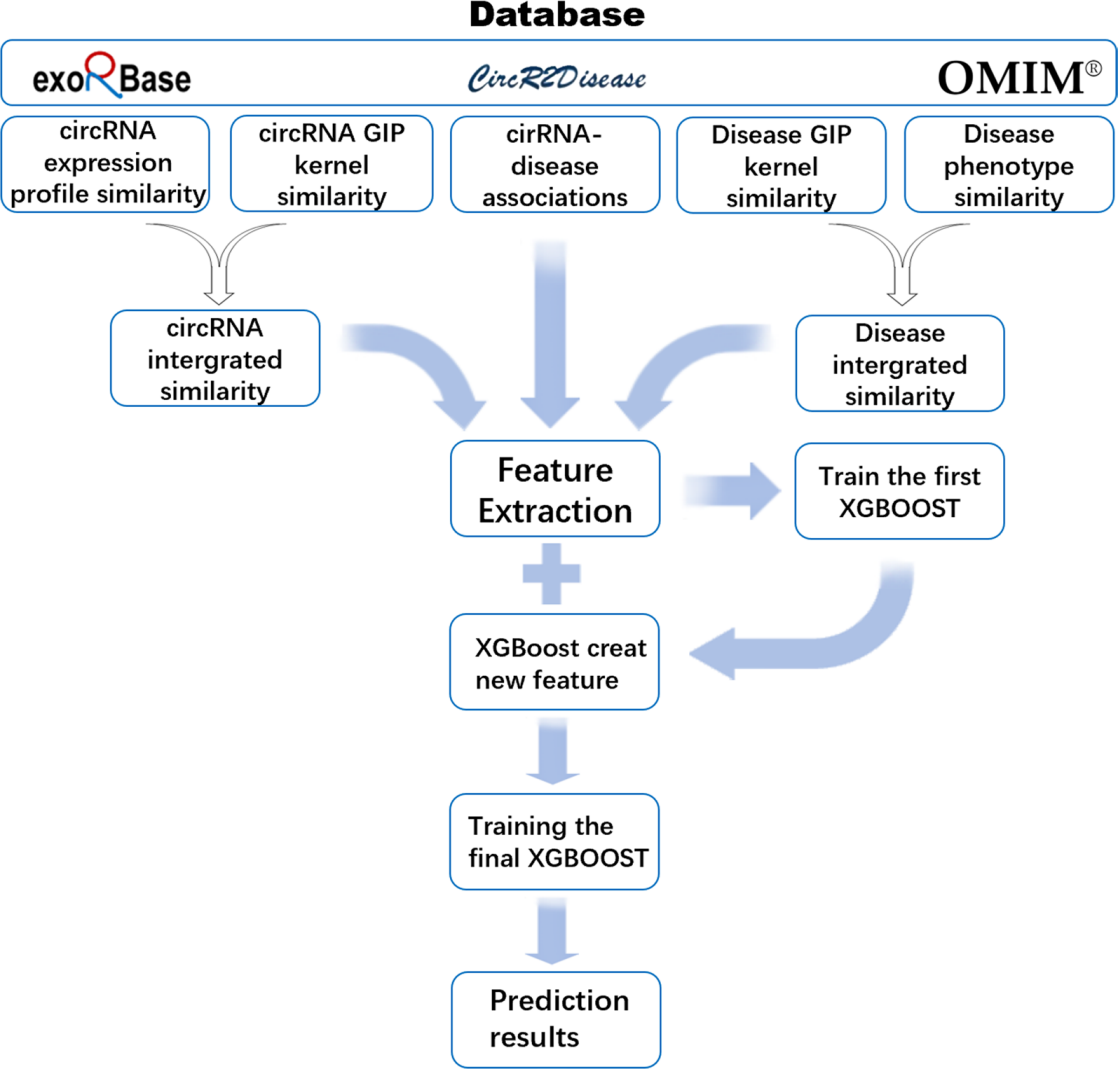
The Flowchart of the XGBCDA method. The XGBCDA method includes the following steps: extract statistical features and graph theory features from an integrated circRNA similarity network, an integrated disease similarity network and circRNA-disease association network; input these original features into the XGBoost classifier to further obtain latent features; integrate the latent features with the original features to train the final XGBoost classifier for predicting circRNA-disease association

In this article, we propose an effective method, named XGBCDA, to predict circRNA-disease associations. Initially, we construct a circRNA similarity matrix composed of circRNA expression profile similarity and Gaussian interaction profile kernel similarity, and a disease similarity matrix composed of disease semantic similarity and Gaussian interaction profile kernel similarity. Besides, we also integrate the circRNA similarity network, the disease similarity network and the known circRNA-disease association network. Then, We utilize the aforementioned data to calculate original features, namely statistical features and graph theory features, and send extracted original features to the XGBoost classifier to obtain latent features. Finally, we input the fused features into the XGBoost classifier again to predict the circRNA-disease association. As a result, our method achieves outstanding performance on the circR2disease dataset, and with the tenfold cross-validation, the area under the curve (AUC) is 0.9860. Figure 1 illustrates the flowchart of our method.

## Methods

### Human circRNA–disease associations

In this study, we obtain human circRNA-disease associations dataset from the CircR2Disease database, including 660 circRNA-disease associations between 604 circRNAs and 88 diseases. CircR2disease provides experimentally verified circRNA-disease associations, which is of great help to our further research in this field. Here, we use adjacency matrix A to represent the circRNA-disease association. If a certain circRNA *c*_*i*_ is related to the disease *d*_*j*_, then we assign the element A(*c*_*i*_, *d*_*j*_) to 1, otherwise to 0.

### circRNA similarity

#### circRNA expression profile similarity

We download 49 human circRNA expression profile data from the exoRbase database [13], whose current version contains 58,330 circRNAs. Then we unify the circRNA id in exoRbase with the circRNA id in the aforementioned circR2disease. Next, we use the person correlation coefficient to calculate the similarity of the expression profile between two circRNAs, represented as element CS_EP(X,Y). If the person correlation coefficient of circRNA X and circRNA Y is higher than the threshold, the element CS_EP(X,Y) is assigned to 1, otherwise 0. In this method, we assign the threshold to 0.4. The similarity of two circRNA is defined as follows:

1 $$CS\_EP\left( {X,Y} \right) = \frac{{\mathop \sum \nolimits_{i = 1}^{n} \left( {X_{i} - \overline{X}} \right)\left( {Y_{i} - \overline{Y}} \right)}}{{\sqrt {\mathop \sum \nolimits_{i = 1}^{n} \left( {X_{i} - \overline{X}} \right)^{2} } \sqrt {\mathop \sum \nolimits_{i = 1}^{n} \left( {Y_{i} - \overline{Y}} \right)^{2} } }}$$

#### circRNA GIP kernel similarity

Based on the hypothesis that similar diseases may be related to similar circRNAs, we calculate the similarity of the Gaussian interaction profile kernel of circRNAs [23]. The Gaussian kernel function is a scalar function that is symmetric along the radial direction and it is widely used in constructing the kernel with eigenvectors [24]. In 1964, Aizermann et al. applied this approach to machine learning to study the potential function method [25]. The specific formula is as follows:

2 $$CS\_GS\left( {c_{i} ,c_{j} } \right) = {\text{exp}}\left( { - \gamma_{c} \left| {\left| {y_{{c_{i} }} - y_{{c_{j} }} } \right|} \right|^{2} } \right)$$

The parameter *γ*_*c*_ has impact on adjusting the calculated kernel bandwidth. Here we define the value of *γ*_*c*_ as follows:

3 $$\gamma_{c} = \frac{{\gamma_{c}^{^{\prime}} }}{{\left( {\frac{1}{{n_{c} }}\mathop \sum \nolimits_{i = 1}^{{n_{c} }} \left| {y_{{c_{i} }} } \right|^{2} } \right)}}$$

where *n*_*c*_ represents the number of all circRNAs.

#### circRNA similarity integration

Finally, we integrate the obtained circRNA expression profile similarity with the circRNA Gaussian interaction profile kernel similarity, using the following formula:

4 $$CS\left( {i,j} \right) = \left\{ {\begin{array}{*{20}c} {CS\_EP\left( {c_{i} ,c_{j} } \right), if CS\_EP\left( {c_{i} ,c_{j} } \right) \ne 0} \\ {CS\_GS\left( {c_{i} ,c_{j} } \right),otherwise} \\ \end{array} } \right.$$

### Disease similarity

#### Disease functional similarity

We gather the phenotypic similarity moment data of diseases from Zhang et al. [17]. And we extract the diseases names from the circRNA-disease association in the circR2disease database and employ them to search for the most similar phenotype ID for each disease within the OMIM database. For the sake of ensuring the accuracy of the data, we delete the diseases that do not match the disease phenotype ID in the OMIM database. Eventually, we collect the qualified phenotypic similarity data of the diseases.

#### Disease GIP Kernel similarity

The computational process of disease GIP kernel similarity is analogous to that of disease Gaussian interaction profile kernel similarity. Based on the hypothesis that similar diseases may constantly be related to similar circRNAs [23], we calculate the kernel similarity of the Gaussian interaction profile kernel of a certain disease by following formula:

5 $$DS\_GS\left( {d_{i} ,d_{j} } \right) = {\text{exp}}\left( { - \gamma_{d} \left| {\left| {y_{{d_{i} }} - y_{{d_{j} }} } \right|} \right|^{2} } \right)$$

The parameter *γ*_*d*_ limits the bandwidth. Here we define the value of *γ*_*d*_ as follows:

6 $$\gamma_{d} = \frac{{\gamma_{d}^{^{\prime}} }}{{\left( {\frac{1}{{n_{d} }}\mathop \sum \nolimits_{i = 1}^{{n_{d} }} \left| {y_{{d_{i} }} } \right|^{2} } \right)}}$$

where *n*_*d*_ represents the number of all diseases.

#### Disease similarity integration

We utilize a similar way, as depicted in the integration of circRNA similarity, to integrate the obtained disease semantic similarity with the disease Gaussian interaction profile kernel similarity by the following formula:

7 $$DS\left( {i,j} \right) = \left\{ {\begin{array}{*{20}c} {DS\_SS\left( {d_{i} ,d_{j} } \right), if DS\_SS\left( {d_{i} ,d_{j} } \right) \ne 0} \\ {DS\_GS\left( {d_{i} ,d_{j} } \right),otherwise} \\ \end{array} } \right.$$

### XGBCDA method

**Fig. 2:**
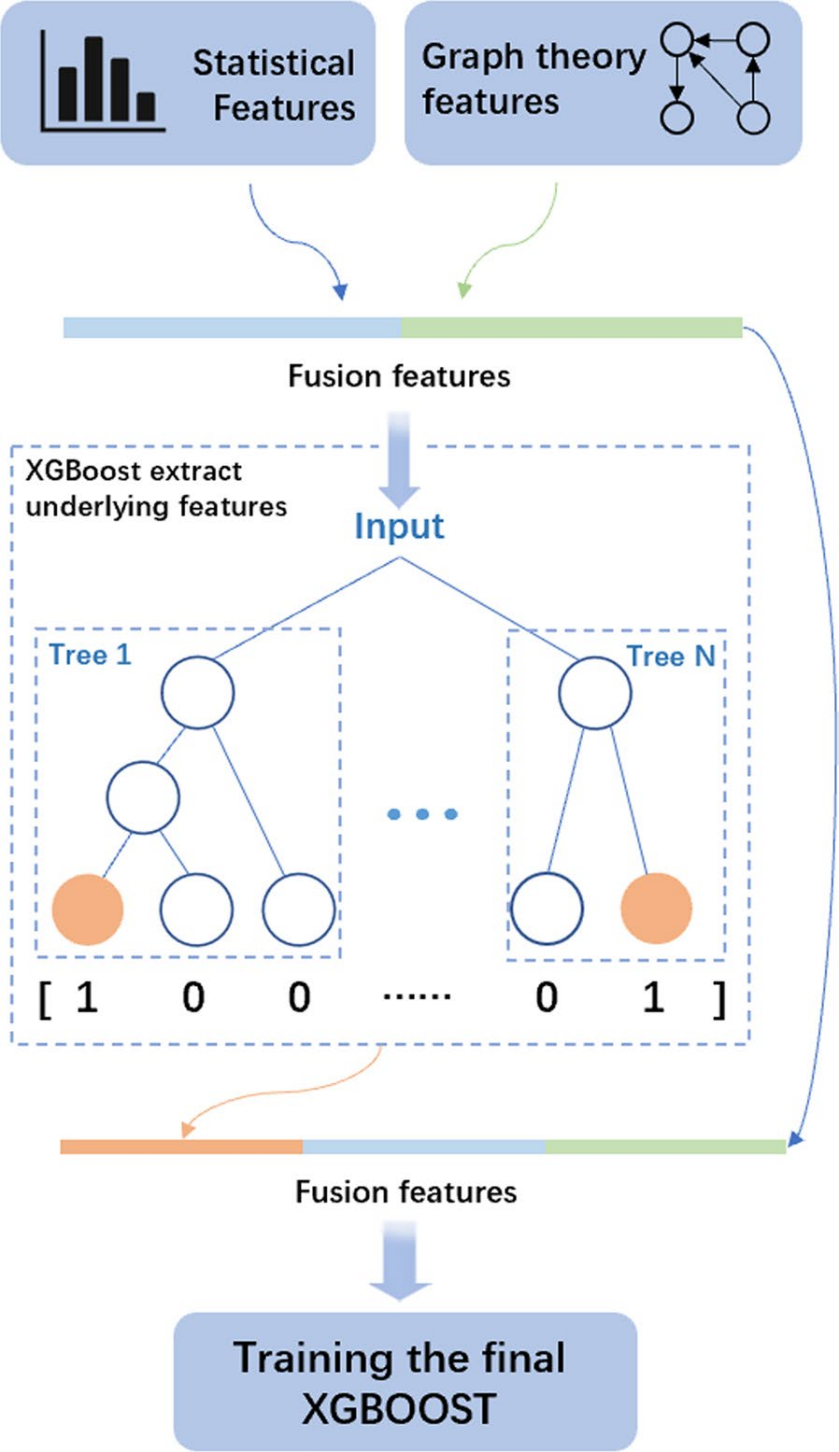
We input the statistical features and graph theory features into XGBoost. The two trees in the figure were learned by XGBoost. For an input sample, if it falls on the first leaf node of the first tree and the second leaf node of the last tree, the new feature vector obtained by XGBoost is [1, 0, 0 … 0, 1]. The first three digits in the vector refer to the three leaf nodes of the first tree, and the last two digits refer to the two leaf nodes of the second tree

In the XGBCDA method, we construct three matrices, the integrated circRNA similarity matrix CS, the integrated disease similarity matrix DS, and the circRNA-disease association matrix A. Inspired by Tong He et al.’s research [26], we calculate the statistical characteristics of each circRNA/disease similarity score, including the histogram distribution and the mean of similarity scores, according to the circRNA similarity matrix CS and the disease similarity matrix DS respectively. Besides, we construct a network whose nodes are circRNA/disease, according to the circRNA/disease similarity matrix. In the network, if the similarity score between two nodes is higher than the average similarity score, then there is an edge between two nodes. We also calculate the number of neighbors that each node has, and nodes’ graph theory characteristics, namely degree centrality, closeness centrality, betweenness centrality. Then, we select the 10 nodes closest to the node’s similarity score as neighbors, and calculate the average and histogram distribution of their similarity scores. In addition, we design a network whose nodes are circRNA and disease, according to the circRNA-disease association matrix A, and use the NMF (Non-Negative Matrix Factorization) algorithm to calculate the latent vector. We then combine the above features to construct a composite feature vector to train the XGBoost model. Subsequently, we use the tree learned by the XGBoost model to form new features. Finally, these new features accompanied with the original features are added to the model for training. After finishing all the procedures, we put the trained XGBoost model into predicting potential circRNA-disease associations. The complete process is illustrated in Fig. 2.

## Results

### Performance evaluation

**Fig. 3:**
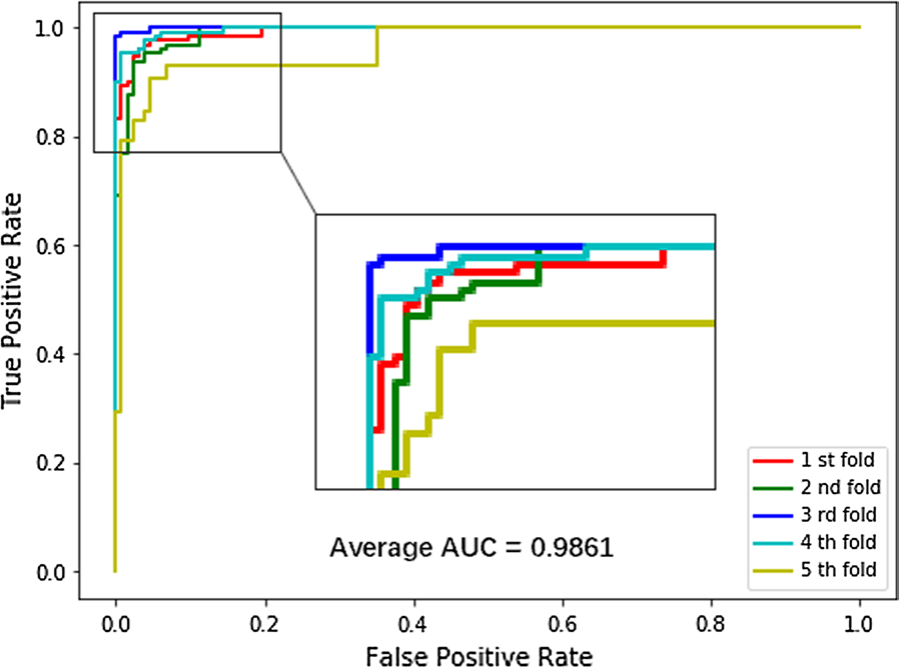
ROC curves from fivefold cross-validation performed with XGBCDA model on circR2Disease dataset

In order to comprehensively assess the prediction performance of our method, we implement the method on the CIRCR2Disease dataset by fivefold cross-validation. Our data set contains positive samples, namely all 660 pairs of known circRNA-disease associations, and negative samples, namely the same amount of unknown associations. Based on the fivefold cross-validation, the area under the curve (AUC) of our method is 0.9935, 0.9913, 0.9996, 0.9968 and 0.9660 respectively, and the average AUC is 0.9861. The experimental results are summarized in Fig. 3.

### Comparison with different classifiers

**Fig. 4:**
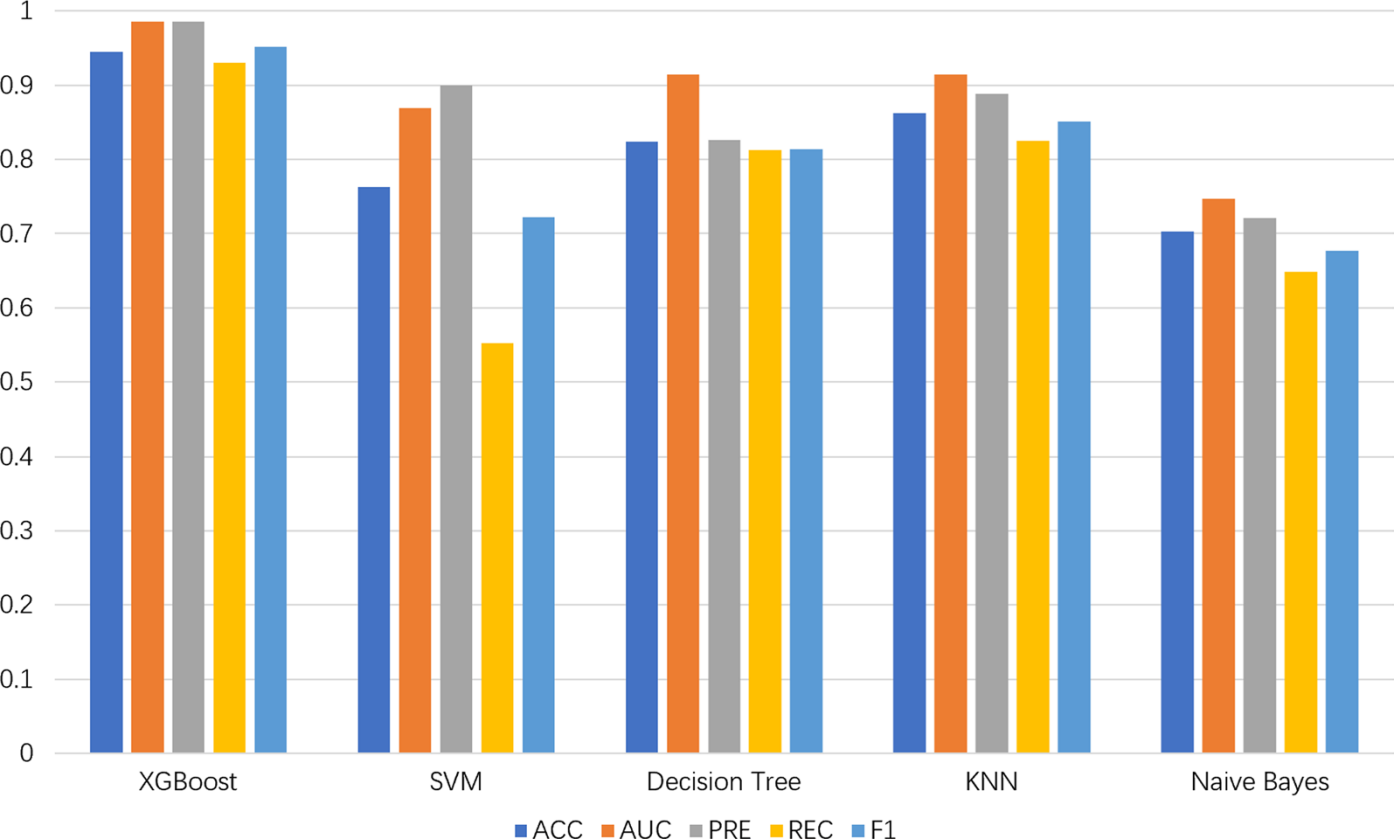
Compare with other classifier models in tenfold cross-validation on the same dataset

To verify the XGBoost classifier’s performance in the model, we compared it with other four popular classifier models(SVM, Decision Tree, KNN, Naive Bayes). These five classifiers all share the same data set, and to ensure the validity of the comparison, we use the default parameters for training and prediction. The evaluation criteria includes accuracy(ACC), Area under the ROC curve(AUC), precision(PRE), recall(REC). With tenfold cross-validation, all parameters of the XGBoost model are ahead of other classifier models’, and the verification results of the remaining four classifier models were shown in Table 1. For an apparent comparison, we present the results of these five models in the form of the histogram. From Fig. 4, it is evident that the XGBoost exhibits the first-rate competence in the evaluation. The comparative experiment results fully prove that the XGBoost classifier is superior to other classifier models in every aspect.

**Table 1:** Compare with other classifier models in tenfold cross-validation on the same dataset

Method	ACC	AUC	PRE	REC	F1
XGBoost	0.9541	0.9860	0.9844	0.9307	0.9517
SVM	0.7626	0.8696	0.8995	0.5519	0.7217
Decision Tree	0.8242	0.9146	0.8266	0.8124	0.8138
KNN	0.8618	0.9147	0.8885	0.8247	0.8509
Naive Bayes	0.7031	0.7469	0.7214	0.6485	0.6766

### Selection of optimal parameter values

In order to further understand the robustness of our proposed method, we analyze the optimal values of 5 parameters in the XGBoost classifier that have the main impact on the performance of tenfold CV, including learning_rate, n_estimators, max_depth, min_child_weight and gamma. We use the cv function in the python package of xgboost to calculate the best values of the learning_rate and n_estimators parameters, which are 0.1 and 463, respectively. We apply the grid search method to determine the parameters max depth and min child weight to be 5 and 4, respectively. We try 5 representative values to test the optimal value of gamma, which are 1e−5, 1e−2, 0.1, 1, 100. Table 2 below proves that 1 is the best value of gamma.

**Table 2:** The tenfold CV prediction performance of various parameter values ranging from 1e−5 to 100 for gamma

gamma	1e−5	1e−2	0.1	1	100
AUC	0.9849	0.9852	0.9860	0.9844	0.9018

### Comparison with other methods

**Fig. 5:**
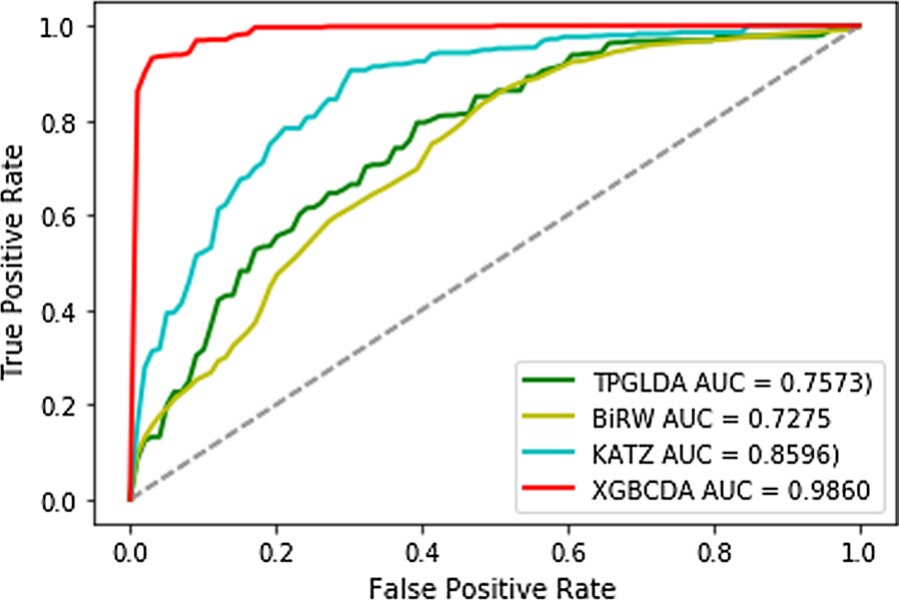
ROC curves of different methods

To thoroughly confirm the best performance of the proposed model, we compare XGBCDA with other state-of-art methods. In comparison with LncRDNetFlow [27], TPGLDA and BiRW [28] and KATZ [29], we use all human circRNA-disease associations in the circR2disease database, defined as positive samples, and the same number of unproven circRNA-disease, defined as negative samples, to form the data set. The Fig. 5 presents that under tenfold cross-validation, the performance of our method significantly exceeds that of the other four methods, and the AUC of our method is 0.9860.

### Latent features extracted from XGBoost

**Fig. 6:**
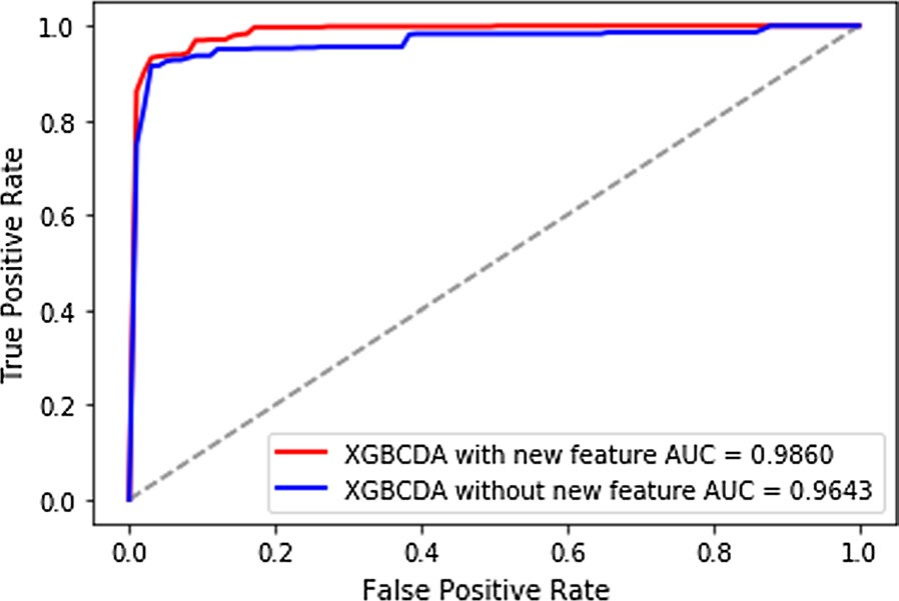
AUC results of XGBCDA with latent feature and that without latent feature under tenfold cross-validations

We compare the model that uses XGBoost to generate new features with the model that does not. XGBoost is also known as eXtreme Gradient Boosting package [30], and has applied to handle multiple tasks, such as regression, classification, and sorting. Furthermore, its advantages involve fast training speed and marvelous prediction performance. Given the aforesaid traits and the work of He et al. [31], we used XGBoost to extract latent features based on original features. We consider each tree as a classification feature and use the leaf index that the instance finally falls into as a value. And the ultimate latent features are coded by 1 Of K coding. Figure 6 depicts that based on tenfold cross-validation, the model using the latent features generated by XGBoost has better performance.

### Case studies

To further evaluate the performance of our method in predicting potential circRNA-disease associations, we select the top 20 associations by prediction scores for verification. The results are presented in Table 3. In addition, we choose three diseases, which are rectal cancer, gastric cancer and cervical cancer, to conduct case studies. We pick 660 known human circRNA-disease associations from circR2Disease as training data. In terms of prediction results, the prediction scores of potential circRNA-disease associations range from 0 to 1, where 1 refers to the highest possibility of the association, and 0 refers to the lowest. In the method, we assume that circRNA-disease associations with a score higher than 0.9 have a high degree of confidence, and we select all circRNA-disease associations, which are not included in the circR2disease database, with predictive scores higher than 0.9 in the three diseases of rectal cancer, gastric cancer and cervical cancer. Among the obtained ten pairs of associations, three pairs of circRNA-disease associations have been confirmed in the literature. However, it is worth noting that this does not mean that the other 7 circRNA-disease pairs must not be related. The results are summarized in Table 4.

**Table 3:** Based on the known association predictions in the circR2disease database, the 20 circRNA-disease pairs with the highest scores

Disease	circRNA	Source
Coronary artery disease	has_circRNA6510_1	CircR2Disease
Coronary artery disease	hsa_circRNA11783_2	CircR2Disease
Coronary artery disease	hsa_circRNA11806_28	CircR2Disease
Osteosarcoma	hsa_circ_0092509	CircR2Disease
Hepatocellular carcinoma	circC3P1	CircR2Disease
Osteosarcoma	hsa_circ_0009910	CircR2Disease
Major depressive disorder	hsa_circ_0001410	CircR2Disease
Major depressive disorder	hsa_circ_0001907	CircR2Disease
Major depressive disorder	hsa_circ_0005620	CircR2Disease
Major depressive disorder	hsa_circ_0056048	CircR2Disease
Major depressive disorder	hsa_circ_0005620	CircR2Disease
Bladder cancer	hsa_circ_0041103	CircR2Disease
Bladder cancer	hsa_circ_0007158	CircR2Disease
Bladder cancer	hsa_circ_0082582	CircR2Disease
Bladder cancer	hsa_circ_0072088	CircR2Disease
Hepatocellular carcinoma	circRNA_000839	CircR2Disease
Bladder cancer	hsa_circ_0061265	CircR2Disease
Hepatocellular carcinoma	hsa_circRNA_104135	CircR2Disease
Primary hepatic carcinoma	hsa_circRNA_100571	CircR2Disease
Coronary artery disease	hsa_circRNA5974_1	CircR2Disease
Osteosarcoma	hsa_circ_0056288	CircR2Disease
Hepatocellular carcinoma	hsa_circ_005075	CircR2Disease

**Table 4:** Validation results of circRNA-disease associations, which are not included in circR2disease, with predicted scores of rectal cancer, stomach cancer, and cervical cancer greater than 0.9 points

Disease	circRNA	Score	Source
Colorectal cancer	has_circ_0000504	0.9990	Unknown
	hsa_circ_0001821	0.979671	PMID: 31616472
Gastric cancer	hsa_circ_0001313	0.990974	PMID: 32253030
	hsa_circ_0001141	0.990562	Unknown
Cervical cancer	hsa_circ_0001649	0.965188	Unknown
	hsa_circ_0001313	0.958534	Unknown
	hsa_circ_0001445	0.942731	PMID: 30575898
	hsa_circ_0001946	0.911142	Unknown
	hsa_circ_0001821	0.903885	Unknown
	hsa_circ_0001141	0.903885	Unknown

## Discussion

We suppose that one of the possible approaches to improve the performance is utilizing other biological information as bridge, given the fact that the researches of the direct association between the circRNA and disease are in the infant stage. For instance, with the growing researches of circRNA-miRNA associations and miRNA-diseases associations, it is worth trying to use miRNA as an intermediary to enhance the performance of our method. Moreover, because the circRNA-RBP data increases exponentially, RBP may be another domain for us to explore.

## Conclusion

In this paper, we proposed an effective method to predict circRNA-disease associations by integrating the semantic similarity of diseases, the similarity of circRNA expression profiles, and the Gaussian interaction profile kernel similarity of circRNA and disease, and using XGBoost to construct latent features. Based on the circR2disease data set, we predict ten pairs of unknown circRNA-disease associations, of which three pairs have been confirmed in the literature. Although our method has achieved extraordinary performance, there is scope for improvement in the future. With the continuous development of ncRNA research by researchers, circRNA-disease associations and lncRNA-disease associations have been gradually discovered, and we can use the valuable information to develop circRNA-disease association predictions.

## Data Availability

The experiment-supported circRNA-disease associations were obtained from circR2disease database(http://bioinfo.snnu.edu.cn/). The code and datasets are available at https://github.com/Q1DT/XGBCDA.

## Abbreviations

circRNAs: Circular RNAs
GIP: Gaussian interaction profiles
LOOCV: Leave-one-out cross validation
fivefold CV: 5-Fold cross validation
tenfold CV: 10-Fold cross validation
OMIM: Online Mendelian Inheritance in Man
AUC: Area under the curve
XGBCDA: Our proposed computational method
XGBOOST: EXtreme Gradient Boosting
