# Correction to: XGBCDA: a multiple heterogeneous networks-based method for predicting circRNA-disease associations

**DOI:** 10.1186/s12920-023-01475-1

**Published:** 2023-03-03

**Authors:** Siyuan Shen, Junyi Liu, Cheng Zhou, Yurong Qian, Lei Deng

**Affiliations:** 1 School of Software, Xinjiang University, Wulumuqi, 830091 China; 2 School of Computer Science and Engineering, Central South University, Changsha, 410075 China

**Correction to: BMC Medical Genomics (2021) 13:196**

10.1186/s12920-021-01054-2

Following publication of the original article [1], it was reported that this article should have been published in Volume 14, Supplement 3.

The details of the supplement in which this article ought to have been published are given below:

About this supplement

This article has been published as part of BMC Medical Genomics Volume 14 Supplement 3 2021: Selected articles from the 19th Asia Pacifc Bioinformatics Conference (APBC 2021): medical genomics The full contents of the supplement are available at https://bmcmedgenomics.biomedcentral.com/articles/supplements/volume-14-supplement-3

The publisher apologizes for any inconvenience caused.

